# Patients undergoing thyroid surgery suffer preoperatively from poor quality of life

**DOI:** 10.1007/s40618-025-02661-x

**Published:** 2025-07-29

**Authors:** Juha Honkanen, Krisztina Molnár, Tuomo Karhumaa, Sanna Kakko, Petri Koivunen, Hanna Franssila, Ulla Koskela, Tiina Erkinaro, Janne Liisanantti

**Affiliations:** Oulu University Hospital and University of Oulu, Kajaanintie 50, Oulu, 90220 Finland

**Keywords:** Thyroid surgery, Quality of life, Thyroid cancer, Thyroidectomy, Lobectomy

## Abstract

**Purpose:**

Thyroid surgery is performed for various indications, including goiter, suspected or diagnosed malignancy, and hyperthyroidism. While previous studies have focused on postoperative quality of life (QoL) improvements, limited data exist on preoperative QoL in different patient groups. This study aimed to assess the preoperative QoL of patients undergoing thyroid surgery and identify risk factors associated with poor QoL. Additionally, we compared the QoL of patients with thyroid cancer or suspected malignancy to those undergoing surgery for non-cancer-related indications.

**Methods:**

This prospective study included 204 patients who underwent thyroidectomy or lobectomy at Oulu University Hospital, Finland, between September 2021 and December 2022. Patients completed preoperative QoL assessments using the RAND-36, ThyPRO-39, Beck Depression Inventory (BDI), and Voice Handicap Index (VHI) questionnaires. Patient characteristics and clinical data were collected from medical records. Statistical analyses included Mann-Whitney U tests, Pearson Chi-square tests, and logistic regression to evaluate differences between patient groups and identify factors associated with poor QoL.

**Results:**

Patients undergoing thyroid surgery, regardless of indication, reported lower QoL compared to the general Finnish population. No significant differences were found between cancer and non-cancer patients in overall QoL scores. Poor QoL was significantly associated with younger age (< 65 years), higher BDI and VHI scores, elevated BMI, and frequent dyspnea.

**Conclusion:**

Preoperative QoL is significantly lower in patients undergoing thyroid surgery compared to the general population, independent of diagnosis. Contrary to our hypothesis, patients with thyroid cancer did not report worse QoL than those with non-malignant thyroid diseases.

## Introduction

Common indications for thyroid surgery include goiter, diagnosed or suspected malignancy, and hyperthyroidism. In Finland, approximately 2,500 thyroid surgeries are performed annually [1], of which about 500 are conducted due to cancer [2]. Accordingly, a significant proportion of annual thyroid surgeries are performed for reasons other than cancer. In a large part of this patient population, particularly among those with goiter, the decision to operate is based on the patient’s symptoms.

Common symptoms of goiter that may lead to thyroid surgery include compressive symptoms (e.g., dyspnea, dysphagia) and, in some cases, cosmetic concerns [12, 13]. Common symptoms of hyperthyroidism such as tremor, palpitations, anxiety, weight loss, diarrhoea, dyspnea, and eye symptoms [16] can also significantly impair patients’ perceived quality of life (QoL) [14, 17].

The existing literature provides limited evidence on the preoperative QoL of patients undergoing thyroid surgery for various diagnoses compared to the general population, as most studies focus on postoperative changes, which have been shown to indicate an improvement in QoL [15, 18, 19]. Notably, no studies have directly compared the preoperative QoL of patients undergoing surgery for thyroid cancer or suspected malignancy with that of patients operated on for non-cancer-related indications.

Understanding preoperative quality of life is especially important in thyroid surgery, as the decision to operate—particularly in benign cases—is often guided by patient-reported symptoms rather than objective clinical necessity. Clarifying the baseline QoL may help distinguish symptoms related to thyroid disease from those potentially linked to coexisting conditions such as depression, enabling more informed decision-making and more realistic expectations for surgical outcomes. For this reason, we focused our study design on preoperative QoL.

The aim of this study was to assess the preoperative QoL of patients undergoing thyroid surgery for various indications and identify risk factors associated with poor QoL. We also aimed to evaluate the differences in QoL before thyroid surgery between patients with diagnosed or suspected cancer and those with other diagnoses. Our hypothesis was that patients diagnosed with cancer perceive their preoperative QoL to be worse compared to those undergoing surgery for other indications.

## Materials and methods

This prospective follow-up study was approved by the ethical committee of the Northern Ostrobothnia hospital district. The study population consisted of patients who underwent either total thyroidectomy or lobectomy at Oulu University Hospital, Finland, between September 2021 and December 2022. A total of 320 patients were operated on during this period. We included in this study those 204 patients from the patient cohort who had completed preoperative QoL questionnaires (RAND-36 and ThyPRO-39, questionnaires explained later).

Data on patients’ general health status and socioeconomic background were collected from their medical records, anaesthesia charts, and laboratory results.

We developed a custom 18-item background questionnaire [att. 1], which included symptom-related questions based on the experience of our unit’s surgeons regarding the symptoms most frequently reported during the preoperative visit. Questions were scored on a scale of 1 to 5, where 1 indicated the absence of the symptom (never) and 5 indicated the constant presence of the symptom (always).

QoL and mood were assessed using the following preoperatively completed patient questionnaires: RAND 36-item health survey 1.0 (RAND-36) [8], Thyroid-Specific Patient-Reported Outcome questionnaire (ThyPRO-39) [9], the Beck Depression Inventory (BDI) [10] and the Voice Handicap Index (VHI) [11].

RAND-36 is a widely used survey instrument for assessing health-related quality of life (HRQoL). It consists of 36 questions that cover eight different domains: physical functioning, role limitations caused by physical health problems, role limitations caused by emotional problems, energy/fatigue, emotional well-being, social functioning, pain and general health perceptions. Each domain is scored from 0 to 100, where 100 represents the highest QoL [8]. A validated Finnish-language translation and age-adjusted reference values for the Finnish population are available [4]. Consistent with previous studies, poor QoL was defined as a score more than − 2 standard deviations (SD) below the age-adjusted reference values in each domain [5–7].

ThyPRO-39 is an international, well-validated, patient-reported questionnaire measuring thyroid related QoL. It consists of 39 questions covering 14 different domains: goiter symptoms, hyperthyroid symptoms, hypothyroid symptoms, eye symptoms, tiredness, vigor, cognition, anxiety, depression, emotional susceptibility, social life, daily life, cosmetic complaints and QoL. Each question is scored on a scale from 1 to 5, where 1 indicates that the patient does not experience the symptom, and 5 indicates that the patient experiences the symptom to a great extent. The higher the score a patient receives in each domain, the greater the severity of symptoms or functional impairment the patient perceives in that specific domain [9].

The BDI is a commonly used 21-item questionnaire assessing the severity of depression. For each question, the patient selects the statement that best describes his/her symptoms from four options related to depressive symptoms. Scores ranging from 10 to 16 indicate mild depressive symptoms, 17 to 29 indicate moderate symptoms, and scores over 30 indicate severe depressive symptoms [10].

The VHI is a patient-reported questionnaire designed to assess the impact of voice-related problems. It consists of 30 questions evenly distributed across three domains: functional, physical, and emotional aspects of voice disorders. The questions were scored on a scale from 0 to 4, where 0 indicates the absence of the symptom, and 4 indicates that the patient always experiences the symptom. The higher the score the patients receive, the more symptoms they report experiencing [11].

The data were analyzed using SPSS software (IBM Corp., IBM SPSS Statistics for Windows, Version 27.0, Armonk, NY, USA). For the first analysis, we divided patients into two groups: those undergoing surgery due to diagnosed or suspected cancer, and those undergoing surgery for non-cancer-related indications. In the second analysis, we divided the cohort into patients with good QoL and those with poor QoL.

Differences between the groups were analyzed using the Mann-Whitney U test for continuous variables and the Pearson Chi-square test or Fisher’s exact test for categorical variables. Logistic regression analysis was used to calculate odds ratios (OR) with 95% confidence intervals (95% CI) for poor preoperative QoL. All the clinically relevant variables with a univariate significance less than 0.1 were included in the model using the enter method. Variables with multivariate significance of less than 0.05 and those with a significant impact on log-likelihood function were retained in the model.

We report continuous data as means with standard deviation (SD) and categorical data as numbers (n) with percentage (%). Two-tailed p values are given.

## Results

The mean age of the patients was 52 years and 78% of them were women. There were no significant differences in patients’ characteristics between patients undergoing surgery due to diagnosed or suspected cancer and those undergoing surgery for non-cancer-related indications, except for slightly lower TSH levels observed in patients having surgery for diagnoses other than malignancy. There was also no difference between the groups in the preoperative use of levothyroxine. In the entire cohort, 15 patients (7.4%) were receiving thyroid hormone replacement therapy prior to surgery (Table 1).

The proportion of patients experiencing poor QoL was comparable in both groups. Also, no differences were observed in the preoperative BDI and VH questionnaires.

In our background questionnaire, patients undergoing surgery for non-cancer-related reasons reported the following preoperative symptoms significantly more often: a sensation of pressure in the neck, pressure in the neck when bending the head downward, shortness of breath with light exertion, shortness of breath while lying on their back, and coughing or throat clearing. Additionally, this group reported significantly poorer sleep quality and greater difficulty working with their hands raised overhead (Table 1).

**Table 1:** Patients’ characteristics

	Cancer *N* = 86	Other than cancer *N* = 118	*P*-value
Age	53.4 (15.9)	50.4 (15.4)	0.134
Female Gender	66 (76.7)	93 (79.5)	0.639
Higher level of education (university of applied sciences or university)	37 (50.0)	43 (45.3)	0.341
Comorbidity			
HTA	31 (36.0)	39 (33.1)	0.656
Stroke	3 (3.5)	7 (5.9)	0.524*
Asthma	10 (11.6)	21 (17.8)	0.226
COPD	2 (2.3)	1 (0.8)	0.574*
MCC	5 (5.8)	2 (1.7)	0.135*
Rheumatoid arthritis	2 (2.3)	8 (6.8)	0.196
Depression	1 (2.3)	0	0.177*
Smoking/snuff	11 (12.8)	25 (21.6)	0.108
Alcohol	44 (51.2)	61 (52.6)	0.841
BMI	28.9 (6.9)	31.6 (22.6)	0.462
Levothyroxine replacement therapy	8 (9.3)	7 (6.0)	0.372
Preop. TSH	2.0 (1.7)	1.6 (2.2)	0.004
Preop. T4V	14.5 (1.9)	15.2 (4.2)	0.248
Poor QoL	40 (46.5)	51 (43.2)	0.641
VH	13.9 (18.7)	16.7 (18.3)	0.054
BDI	9.8 (7.6)	10.1 (8.2)	0.918
Sensation of pressure in the neck	2.7 (1.2)	3.2 (1.3)	0.013
Pressure in the neck when bending the head downward	2.4 (1.4)	3.2 (1.4)	< 0.001
Good quality of sleep	3.6 (1.1)	3.3 (1.0)	0.005
Shortness of breath with light exertion	1.9 (1.0)	2.6 (1.4)	< 0.001
Shortness of breath when lying on the back	1.7 (1.1)	2.4 (1.2)	< 0.001
Coughing or throat clearing	2.9 (1.1)	3.2 (1.2)	0.022
Ability to work with hands raised overhead	4.0 (1.0)	3.4 (1.2)	< 0.001

*Fisher’s Exact

**Fig. 1:**
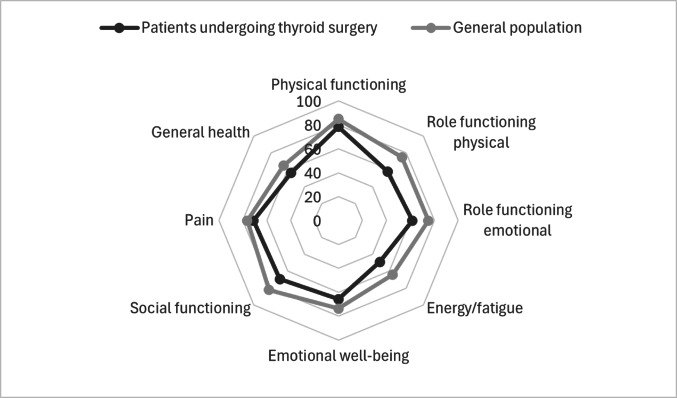
RAND-36 scores in all patients undergoing thyroid surgery compared to the general Finnish population

In the RAND-36 QoL questionnaire, there were no statistically significant differences between thyroid cancer patients and those with other diagnoses in any of the test domains (Table 2). Compared to the general Finnish population, all patients undergoing thyroid surgery, regardless of the underlying cause, reported lower scores in all domains (Fig. 1).

**Table 2:** RAND-36

	Cancer *N* = 86	Other than cancer *N* = 118	*P*-value	General population*
Physical functioning	80.6 (23.5)	76.9 (21.3)	0.056	84.9 (20.1)
Role functioning physical	58.8 (41.3)	57.4 (39.4)	0.702	74.8 (35.5)
Role functioning emotional	55.7 (41.6)	65.8 (39.3)	0.086	75.0 (36.4)
Energy/fatigue	50.5 (23.5)	47.8 (24.1)	0.449	64.0 (22.4)
Emotional well-being	64.8 (19.7)	66.5 (19.7)	0.499	73.7 (19.7)
Social functioning	70.8 (23.8)	68.3 (27.2)	0.683	82.1 (23.2)
Pain	73.3 (23.6)	69.4 (25.3)	0.242	76.2 (24.0)
General health	57.3 (21.8)	55.7 (21.0)	0.573	65.0 (19.8)

*Aalto A-M, Aro AR and Teperi J: Rand-36 as a measure of health-related quality of life. Reliability, construct validity and reference values in the Finnish general population. Helsinki: National Research and Development Center for Welfare and Health. (Research #101 In Finnish, summary in English), 1999

We also conducted an additional analysis comparing patients undergoing surgery for cancer, goiter, and hyperthyroidism. In this comparison, a statistically significant change in physical functioning was observed in the RAND-36, with goiter patients reporting worse physical functioning than the other groups. No significant differences were found in other domains (data not shown).

In the ThyPRO-39 questionnaire, patients undergoing surgery for non-cancer-related reasons reported significantly more symptoms of goiter, hyperthyroidism, and cosmetic complaints, and their self-reported QoL was lower than that of the cancer surgery group (Table 3).

**Table 3:** ThyPRO-39 scores

	Cancer *N* = 86	Other than cancer *N* = 118	*P*-value
Goitre symptoms	48.9 (22.7)	60.1 (24.3)	< 0.001
Hyperthyroid symptoms	40.6 (18.4)	47.3 (21.0)	0.031
Hypothyroid symptoms	47.6 (17.6)	50.5 (20.2)	0.415
Eye symptoms	48.2 (21.9)	52.9 (22.2)	0.114
Tiredness	65.0 (21.5)	70.3 (22.0)	0.071
Vigor	50.2 (21.2)	51.4 (22.0)	0.856
Cognition	49.7 (23.1)	53.8 (22.9)	0.178
Anxiety	52.0 (21.3)	50.0 (30.3)	0.191
Depression	55.0 (15.7)	52.3 (16.6)	0.245
Emotional susceptibility	60.4 (16.8)	62.4 (17.3)	0.352
Social life	35.7 (18.2)	37.1 (18.6)	0.606
Daily life	40.4 (19.9)	42.8 (21.2)	0.480
Cosmetic complaints	32.4 (17.7)	40.6 (21.2)	0.002
Quality of life	53.0 (26.0)	64.9 (26.1)	0.002

When the patient cohort was divided into groups based on good versus poor QoL, higher preoperative VH and BDI scores, as well as a higher BMI, were associated with decreased QoL. Patients with poor QoL had an average BDI score of 15, indicating mild depression (Table 4).

**Table 4:** Quality of life

	Good QoL *N* = 113	Poor QoL *N* = 91	*P*-value
Age > 65 years	36 (31.9)	19 (20.9)	0.079
Female Gender	84 (75.0)	75 (82.4)	0.202
ASA-classification 3–5	18 (16.1)	24 (26.4)	0.072
Cancer diagnosis	43 (39.8)	40 (46.0)	0.386
Basedow diagnosis	22 (20.4)	16 (18.4)	0.729
Goiter diagnosis	43 (39.8)	31 (35.6)	0.549
VH	11.4 (15.7)	20.7 (20.3)	< 0.001
BDI	6.1 (4.4)	14.7 (8.8)	< 0.001
Age	53.0 (16.7)	50.0 (14.1)	0.181
BMI	27.9 (5.6)	33.7 (25.7)	0.004
Preop. TSH	1.82 (2.34)	1.68 (1.57)	0.624
Preop. T4V	14.9 (3.6)	14.8 (3.2)	0.758
Preop. Alb	39.2 (3.0)	41.0 (1.2)	0.192

In multivariate modeling, factors such as age under 65, regular shortness of breath, and BDI scores over 9 were found to increase the risk of poor QoL. A cancer diagnosis did not significantly worsen the patient’s QoL (Table 5). In patients with diagnoses other than cancer, QoL was not dependent on whether the indication for surgery was goiter or hyperthyroidism.

**Table 5:** Poor quality of life

	OR (95% CI)	*P*-value
Preop. BDI scores > 9	8.2 (4.1–16.6)	< 0.001
Regular shortness of breath	4.1 (1.3–12.8)	0.013
Age < 65 years	3.2 (1.3–7.6)	0.010
ASA 3–5	2.5 (1.0-6.4)	0.059
Regular somnipathy	2.0 (1.0-4.1)	0.050
Cancer diagnosis	2.0 (1.0–4.0)	0.066

## Discussion

The main finding of this study is that regardless of the specific thyroid disease diagnosis, patients undergoing thyroid surgery report significantly worse QoL compared to the general Finnish population. Factors predisposing to poor preoperative QoL in this study included age under 65, BDI scores over 9, regular shortness of breath, higher VH scores, and higher BMI. Contrary to our hypothesis, the QoL in patients diagnosed with cancer did not differ from that of patients undergoing surgery for other indications.

This is the first study to assess the QoL of patients undergoing thyroid surgery for various indications and compare it to that of the general population. Additionally, no previous studies have compared the QoL of patients operated on due to thyroid cancer or suspected malignancy with that of patients undergoing thyroid surgery for other diagnoses.

Cramon et al. have previously compared the QoL of patients with nontoxic goiter to that of the general population and found it to be significantly lower. The same study indicated that treatment for goiter (including also thyroid surgery as an option) improved the QoL six months after treatment to some extent, but not to the level of the general population [14]. Liu et al. (2024) investigated the QoL of patients undergoing thyroidectomy due to diagnosed or suspected thyroid cancer and found that the preoperative QoL in this patient group was generally quite good [3].

Concerning the impact of other non-malignant diseases of the head and neck region on preoperative QoL, the literature includes studies on conditions such as nasal polyposis and chronic rhinosinusitis. Deeba et al. investigated the QoL of patients undergoing surgery for nasal polyposis, using the SF-36 measure both pre- and postoperatively. The RAND-36 measure used in the present study comprises exactly the same questions as the SF-36, with only minor differences in the scoring of two subscales (Pain Scale and General Health Scale). However, in the study by Deeba et al., patients scored better on all matching dimensions of the measures used compared to our thyroid patients, and their preoperative QoL was better than that of our patients [23].

Nilsen et al. investigated the preoperative QoL in patients undergoing surgery for chronic rhinosinusitis using the same SF-36 QoL measure. Compared to our patient cohort, the patients with chronic rhinosinusitis experienced greater impairments in emotional well-being and social functioning, while their emotional role functioning was better preserved [24]. Similar to patients undergoing thyroid surgery, both patients with nasal polyposis and those with chronic rhinosinusitis seem to have a significantly lower QoL than the general population.

Several studies have shown that patients undergoing surgery due to cancer or suspected malignancy exhibit higher rates of depression compared to the general population [20–22]. However, a surprising finding in our study was that even thyroid patients undergoing surgery for non-cancer-related indications scored on the BDI within the range indicative of depression, and these scores did not differ significantly from those of patients operated on due to diagnosed or suspected cancer. On the other hand, only one patient in our cohort who underwent surgery due to cancer or suspected cancer had a diagnosed depression, and none of the patients operated on for other indications had a depression diagnosis. We can only speculate on the extent of undiagnosed depression in this patient group and its potential impact on symptoms experienced and QoL perceived.

In cases of diagnosed or suspected thyroid cancer—as well as in Graves’ disease—the indication for surgery is typically clear, regardless of preoperative quality of life (QoL). In many other cases, particularly in patients with goiter, the decision to proceed with surgery is largely influenced by patient-reported symptoms. In such situations, the primary goal is symptom relief through surgical intervention; however, this also carries a risk of potential complications. Assessing preoperative QoL is clinically relevant, as it can inform surgical decision-making and help identify patients who may benefit from psychological support prior to the procedure. Furthermore, understanding a patient’s baseline well-being may help establish more realistic expectations for postoperative outcomes.

### Limitations

A common limitation of QoL studies is how we define good versus poor QoL. In our study, we used the RAND-36 survey for this definition, but other measures are also widely used. We can also question whether a symptom-based survey is even suitable for determining QoL, as symptoms do not necessarily correlate with the patient’s perceived satisfaction. A patient suffering from severe symptoms may not necessarily feel that their QoL is diminished.

In our cohort, we had a total of 320 patients, of whom 204 (64%) responded to the preoperative QoL survey. We are satisfied with the response rate; however, a higher response rate would have strengthened our results.

## Conclusion

Patients undergoing thyroid surgery report a lower preoperative QoL compared to the general population, regardless of the specific thyroid disease diagnosis. Factors associated with poor QoL include age under 65 years, higher scores on the BDI and VH questionnaires, higher BMI, and the patient’s experience of regular shortness of breath. Suspected or diagnosed cancer did not significantly worsen the patients’ QoL.

## Appendix: Attachment 1

### Background questionnaire for participants in the study on Quality of life after thyroid surgery

#### Instructions

Circle the answer that reflects how often you feel the same way.

1 = Never

2 = Rarely

3 = Sometimes

4 = Almost always

5 = Always.

**Gender**: ___________________

**Age**: _______________________

1. Do you smoke (including e-cigarettes) or use snuff?
  Yes / No.
2. Do you consume alcohol?
  Yes / No
  If yes, how many drinks per week? _______ drinks.

(1 drink is, for example, 1 bottle of beer or cider, 1 glass (12 cl) of wine, or 1 shot of whiskey)

3. Do you feel pressure around your neck?
  1 2 3 4 5.
4. Do you feel pressure around your neck when you bend your head down?
  1 2 3 4 5.
5. Have you been able to wear necklaces?
  1 2 3 4 5.
6. Have you been able to wear clothes with a high collar?
  1 2 3 4 5.
7. Do you sleep well?
  1 2 3 4 5.
8. Have you been able to sleep on your back?
  1 2 3 4 5.
9. Do you need to find a comfortable position before going to sleep?
  1 2 3 4 5.
10. Do you experience shortness of breath with light exertion (walking, normal household chores)?
  1 2 3 4 5.
11. Do you experience shortness of breath when lying on your back?
  1 2 3 4 5.
12. Do you have difficulty swallowing?
  1 2 3 4 5.
13. Do you often choke on food or drinks?
  1 2 3 4 5.
14. Is it difficult for you to swallow large pills or coarse food?
  1 2 3 4 5.
15. Do you experience coughing or throat clearing?
  1 2 3 4 5.
16. Do you have a hoarse voice?
  1 2 3 4 5.
17. Can you work with your hands raised above your head?
  1 2 3 4 5.
18. Have your thyroid function levels been tested?
  Yes / No.

What is your most recent thyroid function test result?

TSH _______ T4V _______.

Date of test: __________________.
